# Impact of Omega-3 Fatty Acid Supplementation in Parenteral Nutrition on Inflammatory Markers and Clinical Outcomes in Critically Ill COVID-19 Patients: A Randomized Controlled Trial

**DOI:** 10.3390/nu16183046

**Published:** 2024-09-10

**Authors:** David Berlana, Raquel Albertos, Raquel Barquin, Alba Pau-Parra, Monica Díez-Poch, Rocío López-Martínez, Cristina Cea, Sergi Cantenys-Molina, Roser Ferrer-Costa

**Affiliations:** 1 Pharmacy Department, Vall d’Hebron Barcelona Hospital Campus, 08035 Barcelona, Spain; 2 Pharmacology, Toxicology and Therapeutic Chemistry Department, Faculty of Pharmacy and Food Sciences, University of Barcelona, 08028 Barcelona, Spain; 3 Intensive Care Unit, Vall d’Hebron Barcelona Hospital Campus, 08035 Barcelona, Spain; 4 Biochemistry Department, Vall d’Hebron Barcelona Hospital Campus, 08035 Barcelona, Spain; 5 Immunology Department, Vall d’Hebron Barcelona Hospital Campus, 08035 Barcelona, Spain; 6 Biochemical Chemistry, Drug Delivery & Therapy (BC-DDT) Research Group, Vall d’Hebron Institut de Recerca (VHIR), 08035 Barcelona, Spain

**Keywords:** parenteral nutrition, COVID-19, SARS-CoV-2, omega-3 fatty acids, immunomodulation, intensive care, C-reactive protein, fish oil, clinical trial, inflammatory markers

## Abstract

The heightened inflammatory response observed in COVID-19 patients suggests that omega-3 fatty acids (O3FA) may confer anti-inflammatory benefits. This randomized, double-blind, single-center clinical trial aimed to evaluate the effect of O3FA supplementation in parenteral nutrition (PN) on inflammatory markers in COVID-19 patients admitted to the intensive care unit (ICU). A total of 69 patients were randomized into three groups: one received standard lipid emulsion, and two received O3FA (Omegaven^®^) at doses of 0.1 g/kg/day and 0.2 g/kg/day, respectively, in addition to Smoflipid^®^. The primary outcomes measured were serum levels of C-reactive protein (CRP) and interleukin-6 (IL-6) on days 1, 5, and 10 of PN initiation. Secondary outcomes included additional inflammatory markers (TNF-α, IFN-γ, IL-1Ra, CXCL10), hepatic function, triglyceride levels, and clinical outcomes such as mortality and length of ICU and hospital stay. Results indicated a significant reduction in CRP, IL-6, and CXCL10 levels in the group receiving 0.1 g/kg/day O3FA compared to the control. Additionally, the higher O3FA dose was associated with a shorter ICU and hospital stay. These findings suggest that O3FA supplementation in PN may reduce inflammation and improve clinical outcomes in critically ill COVID-19 patients.

## 1. Introduction

Severe acute respiratory syndrome coronavirus 2 (SARS-CoV-2) is the etiological agent of the coronavirus disease 2019 (COVID-19) [1]. COVID-19 symptoms in patients range from asymptomatic to severe. Severe complications can overstimulate the host immune system, leading to a “cytokine storm”. Critically ill patients with severe acute respiratory distress syndrome and COVID-19 infection often exhibit a hyper-inflammatory state accompanied by a “cytokine storm”. The “cytokine storm” is defined as the overactivated and uncontrolled immune response caused by SARS-CoV-2 infection, leading to severe symptoms in patients, such as severe pneumonia, hypoxemia, and respiratory distress [2,3,4].

Cytokines are signaling molecules with pro- and anti-inflammatory responses that, in a balanced situation, contribute to a healthy immunologic response. However, when a cytokine storm occurs, the proinflammatory cytokine levels increase excessively, leading to clot formation, a decrease in arterial pressure, and organ failure [2,5]. Additionally, the inflammation resolution process is mainly controlled by a group of molecules called specialized pro-resolution mediators (SPMs) [6,7]. These SPMs participate in the neutralization of the proinflammatory cytokines and removal of other remnants, restoring normal structure and tissue homeostasis [6,8]. SPMs have been shown to decrease inflammation and stimulate wound healing and tissue regeneration in experimental models. SPMs are synthesized from long-chain polyunsaturated fatty acids (PUFAs) such as eicosapentaenoic acid (EPA) and docosahexaenoic acid (DHA), both from the omega-3 family. Moreover, SPMs such as maresins and protectins are derived from DHA, whereas resolvins (other SPMs) are derived from both DHA and EPA. The administration of omega-3 fatty acids (O3FA), composed of EPA and DHA, can reduce proinflammatory cytokine production and regulate eicosanoid synthesis derived from arachidonic acid (ARA), which are mediators of inflammation [6,8,9]. Additionally, O3FAs lead to the production of SPMs, which have anti-inflammatory properties [7,10,11].

COVID-19 pathogenesis is complex, involving four main stages typical of acute viral infections: (1) viral invasion and replication, focusing on the respiratory and gastrointestinal epithelium; (2) disruption of barrier functions at inflammation sites, caused by dysregulated innate and adaptive immune responses; (3) systemic spread of the virus, leading to immune dysregulation and increased risk of severe complications; and (4) long-term consequences, including persistent viral presence in the body. Respiratory distress from SARS-CoV-2 is driven by an inflammatory response, primarily due to protease and free radical damage from leukocytes, along with an excess of inflammatory mediators [3,4,12]. The pathogenesis of COVID-19 indicates that adequate nutritional status is a critical factor in moderating susceptibility to and progression of the illness [13]. The method of delivering nutritional support is closely related to the respiratory autonomy of the COVID-19 patient. For critically ill patients, such as SARS-CoV-2 patients, who cannot be fed orally, enteral nutrition should be administered. Parenteral nutrition (PN) should be considered when EN is not indicated or unable to meet nutritional requirements [14].

**Figure 1:**
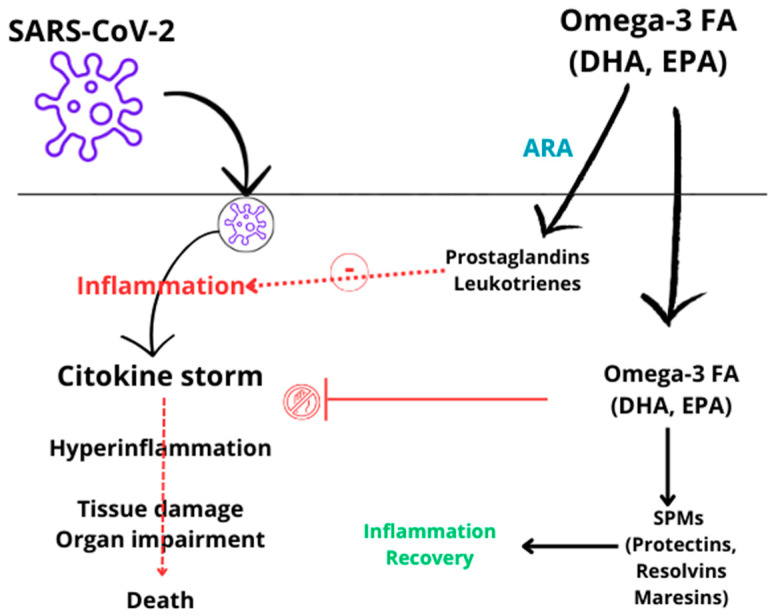
Potential actions of O3FA in pathogenesis of COVID-19.

PN supplemented with O3FA is recommended in critically ill patients, mainly because of its anti-inflammatory properties [15]. O3FAs may provide clinical benefits to critically ill patients, including those suffering from COVID-19, by modulating key pathways involved in inflammation, immune response, and resolution of inflammation [10,16,17]. These compounds are known to inhibit the cellular expression of pro-inflammatory cytokines and chemokines, and they can also be metabolized into SPMs, that actively resolve inflammation [7,10,18]. As mentioned above, SARSCoV-2 causes an increase in cytokines and inflammatory markers like C-reactive protein (CRP). Higher serum levels of inflammatory markers (such as CRP and interleukin [IL] 6) have been associated with disease severity. Therefore, using O3FA may provide potential benefits by reducing the intensity of the inflammatory response of the cytokine storm in COVID-19 [10,18]. Given this potential benefit, we aimed to study the effect of O3FA supplementation in patients with PN on inflammatory markers in critically ill patients with COVID-19. Figure 1 shows the potential actions of O3FA in the pathogenesis of COVID-19.

## 2. Materials and Methods

This double-blind, single-center, randomized clinical trial compared the effects of parenteral O3FA supplementation in ICU patients with SARS-CoV-2 treated with PN. The main objective of this study was to establish whether the administration of PN supplemented with O3FA (Omegaven^®^, Fresenius-Kabi, Bad Homburg, Germany), added to a standard fish-oil-enriched lipid emulsion (LE-Smoflipid^®^, Fresenius Kabi, Bad Homburg, Germany) for 4 to 9 days in critically ill patients with COVID-19, was effective in reducing inflammation, measured using serum concentration of CPR and IL-6, compared to the administration of PN with standard available LE. Additionally, the study aimed to assess whether higher doses of O3FA are more effective in reducing levels of CRP and IL-6. To evaluate this, two different doses of O3FA were administered (0.2 g/kg/day versus 0.1 g/kg/day of fish-oil LE [Omegaven^®^]). 

The Ethics Committee of the Vall d’Hebron Barcelona Campus Hospital approved the study (EudraCT number 2020-000705-86; registered at REEC protocol code 01-2020). Due to the exceptional situation of the COVID-19 pandemic, oral informed consent was obtained from patients’ relatives. Patients who met the following criteria were enrolled: adults (>18 years); admitted to ICU due to COVID-19 pneumonia; starting PN; and provided informed consent. Patients were excluded based on the following reasons: <18 years old; pregnancy; allergy to any of the lipid emulsion components; liver transplantation; or if intravenous lipid administration was contraindicated.

No changes were made to clinical practice in nutritional assessment or PN prescription as a result of inclusion in the study. PN was prepared at the Pharmacy Department regardless of the lipid type. The PNs were delivered ready for administration and stored according to the regular procedures of our hospital. After obtaining informed consent, the patients recruited were randomized into three groups (1:1:1):

1. Group I received the standard LE according to hospital protocol. Since we use different kinds of multichamber-bag (MCB) PNs, they may receive different amounts of O3FA included in the regular LE.
2. Group II received 0.1 g/kg/day of fish oil (Omegaven^®^) plus the amount of LE containing LC/MCT/omega-9 and O3FA (Smoflipid^®^) to reach the total amount of lipid prescribed.
3. Group III received 0.2 g/kg/day of fish oil (Omegaven^®^) plus the amount of LE containing LC/MCT/omega-9 and O3FA (Smoflipid^®^) to reach the total amount of lipid prescribed.

As described above, patients included in Group I received compounded PN with Smoflipid^®^ as LE or regular available MCBs. The MCBs available in our formulary included the following LEs: LCT (Olimel^®^, Baxter, Deerfiel, IL, USA); LCT/MCT/O3FA (Omegaflex^®^, BBraun, Melsungen, Germany); and LCT/MCT/omega-9/O3FA (Smofkabiven^®^, Bad Homburg, Germany). All enrolled patients were randomly assigned to the corresponding intervention group by the pharmaceutical researcher using an allocation sequence (computer-generated random numbers). The sample size was calculated based on prior IL-6 data, where the O3FA administration group had a mean value of 392.53 (526.30 standard deviation [SD]), whereas the mean value of the control group was 31.58 (36.44) [19]. Considering 95% accuracy and 90% power, a sample size of 23 for each group was calculated.

In our hospital, CRP and IL-6 levels were routinely measured in patients with COVID-19. However, the other inflammatory parameters (tumor necrosis factor alpha [TNF-α], interferon gamma [INF-γ], cytokine IL-1Ra, and the chemokine CXCL10) were determined via an Enzyme-Linked Immunosorbent Assay (ELISA) after samples were aliquoted and stored frozen at −80 °C until analysis. Variables were recorded at the time of recruitment (day 1), day 5, and day 10 of the PN initiation. Variables on day 1 provided information about the basal inflammatory status of the patients before PN. Therefore, blood samples on day 1 were taken before PN initiation. As the main outcome is the evolution of inflammatory parameters, CRP and IL-6 on day 1 were compared with those obtained on day 5 and day 10. Similarly, inflammatory parameters related to secondary outcomes (TNF-α, INF-γ, IL-1Ra, and CXCL10) and biochemical parameters (TG, hepatic parameters: total bilirubin [TB], alanine-amino-transferase [ALA], aspartate-amino-transferase [AST], gamma-glutamyl-transferase [GGT], and alkaline phosphatase [AP].) on day 1, day 5, and day-10 were also compared. All patients were followed up until discharge (deceased or alive). Due to the high rate of hospitalization during the COVID-19 pandemic, patients were either discharged or moved to other hospitals or nursing home facilities. Therefore, all known discharge statuses, either during hospitalization in VHBCH or subsequent hospitalization after being moved, were included. Mortality, ICU length of stay (LOS), and hospital LOS were assessed until final discharge (ICU and hospital LOS were reported for survivors). Demographics and clinical and analytical data generated following normal clinical practice were collected and recorded.

The evolution of inflammatory and hepatic parameters, triglycerides, ICU LOS, and hospital LOS grouped into three groups were compared using the analysis of variance (ANOVA) or Kruskal–Wallis test for nonparametric tests. The chi-square test or Fisher’s exact test was used for categorical variables. Values collected from the same patient were compared using ANOVA for repeated measures, paired t-test, or Wilcoxon signed rank test for nonparametric tests (within subjects’ tests). Similarly, baseline characteristics grouped into the three groups were also compared using the ANOVA or Kruskal–Wallis test for continuous variables, and the chi-square test or Fisher exact test for categorical variables. Stepwise multivariate analyses were performed to study risk factors associated with mortality (logistic regression) and those associated with ICU stay and LOS (linear regression). Both intention-to-treat (ITT) analysis and per-protocol (PP) analysis were conducted. The full model included the intervention group, age, and Acute Physiology and Chronic Health Disease Classification System II (APACHE II) score for mortality. For ICU and hospital LOS, the model included the intervention group of or the mean amount of O3FA received, age, and APACHE II score: additionally, for mortality and LOS models, the variables that showed differences between groups were also included in the full model. The best models were selected according to Akaike’s Information Criterion. Statistical analyses were performed using Stata^®^ software, release 16.0 (Stata Corporation, Lakeway, TX, USA), and statistical significance was set as *p* < 0.05.

## 3. Results

**Figure 2:**
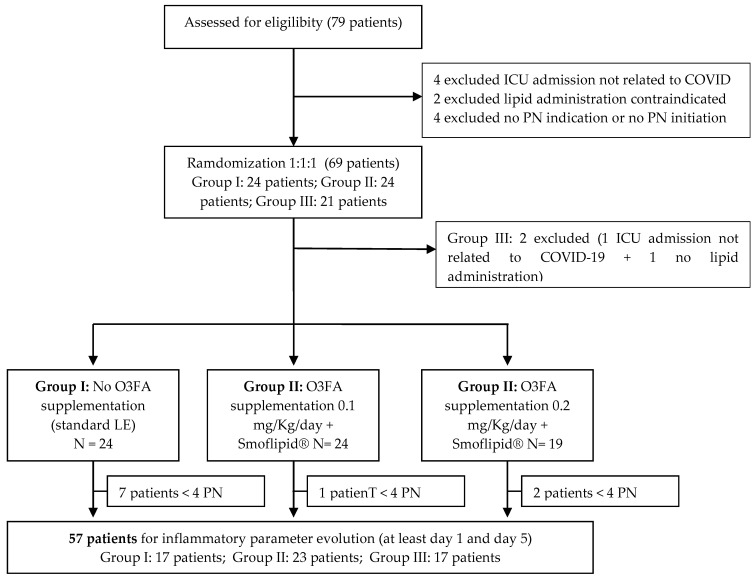
Flowchart of patients randomized in the study and follow-up.

From April 2020 to June 2022, 69 subjects were enrolled in our study: 24 in the control group (Group I) and 45 in the O3FA groups (24 in Group II and 21 in Group III). Two patients from Group III were excluded from the study because they did not meet the inclusion criteria. The study was stopped in June 2022 due to a significant decline in COVID-19 hospitalizations and the use of PN in these patients. Of the remaining 67 patients, 12 did not complete at least 4 days of PN required to assess inflammatory markers, hepatic parameters, and triglyceride levels (day 1 and day 5). Therefore, 56 patients were included in the assessment of these parameters. Figure 2 shows the flow chart.

Baseline parameters, except for CRP and IL-6 levels, did not show significant differences between Groups II and III (Table 1). Regarding the main outcome, the within-subject analysis of CRP and IL-6 levels showed a significant decrease in patients allocated to Group II. Similarly, patients in Group II showed a significant decrease in interleukin CXCL10. Groups II and III did not show significant variation in IL-1Ra levels, whereas patients in Group I showed a significant increase (Table 2). However, due to the lack of initial funding, samples were stored frozen for an extended period. Unfortunately, during this long period, 46 samples intended to determine secondary inflammatory parameters (TNF-α, INF-γ, IL-1Ra, and CXCL10) from 26 patients were damaged due to problems with the freezing process. With regards to differences between groups on day 5 and day 10, no significant differences were observed among the parameters assessed (inflammatory markers, hepatic function, and TG: see Supplementary Table S1).

Regarding PN use, the duration of PN in Group I was longer than in the other two groups, but this difference was not significant (Table 3). The total energy and lipids administered, up to day 5 and day 10, did not show differences between groups. As expected, there were significant differences between groups regarding the amount of O3FA administered. Additionally, univariate analysis showed significantly lower hospital and ICU LOS in patients allocated to Group III (Table 3). Similarly, patients in Group II showed shorter ICU and hospital LOS compared to Group I, but this difference was not significant (*p* = 0.07, Table 3).

Regarding hepatic parameters, no differences were found between days 1 and 5 or days 1 and 10, except for GGT in Group III between days 1 and 5 (180.18 [131.96] vs. 317.59 [348.30], *p* < 0.05—Table 4). Regarding triglyceride levels, only Group II showed a significant difference between days 1 and 10 (297.28 [145.95] vs. 244.22 [95.46], *p* < 0.05—Table 4). Hypertriglyceridemia (>400 mg/dL) developed in 10 patients (58.8%) in Group I, 12 patients (52.2%) in Group II, and six patients (35.3%) among patients receiving >3 days of PN.

Overall, in-hospital mortality was 33.3% (23 patients). There were no differences in mortality between groups, but shorter ICU and hospital stays were observed in patients in Groups II and III (Table 3). Factors associated with mortality and ICU and hospital LOS are presented in Table 5 (PN days and baseline levels of CRP and IL-6 were included in the full model for both ITT and PP). Only the APACHE II score and baseline IL-6 level were included in our cohort’s final model regarding mortality. With regard to ICU LOS, O3FA addition in Groups II and III, or the total amount of O3FA, was included in both final models (ITT and PP), along with the APACHE II score. Similarly, for hospital LOS, the addition of O3FA (in Groups II and III) and the total amount of O3FA were included in both final models, but not the APACHE II score (Table 5).

## 4. Discussion

Our study, conducted as a randomized controlled trial, investigated the effects of adding O3FA to regular fish-oil-enriched LE in critically ill SARS-CoV-2 patients receiving PN. This study showed that O3FA supplementation contributes to suppressing excessive inflammatory responses. However, this suppression may be dose-dependent, as the higher dose of O3FA tested (0.2 g/kg/day plus Smoflipid^®^) did not achieve the same results as the lower dose (0.1 g/kg/day plus Smoflipid^®^). Nonetheless, our findings suggest that higher doses of O3FA in PN may reduce ICU stays and overall hospitalization in critically ill SARS-CoV-2 patients.

Our study demonstrated that SARS-CoV-2 patients receiving O3FA-supplemented PN experienced a decrease in CRP and IL-6 levels, consistent with previous studies [20,21,22,23]. In line with our findings, a recent meta-analysis assessing the use of O3FA in COVID-19 revealed a significant decrease in CRP serum levels [24]. Similarly, our results showed a significant reduction in IL-6 in COVID-19 patients receiving supplemented O3FA PN, whereas COVID-19 patients receiving regular ILE did not. However, a recent meta-analysis failed to show any effect of O3FA on IL-6 among COVID-19 patients [25]. Our results also showed that higher O3FA supplementation in PN did not significantly impact CRP or IL-6 serum levels, whereas lower O3FA PN supplementation did. These inconsistent results might be due to the small sample size and the differences in timing. Patients in Group III showed significantly lower CRP and IL-6 levels on day 1 compared to Group II, suggesting a different timing of the immune response. Timing is crucial due to the complexity of the cytokine storm and the dynamic nature of the immune response [26]. Considering these points and the short time of observation (4 and 9 days), this may explain the lack of effect on CRP and IL-6 in Group II patients.

Studies on O3FA supplementation in COVID-19 patients are heterogeneous, varying in patient population (e.g., mild to moderate COVID-19, ambulatory) and route of administration (enteral or parenteral) [20,21,22,23,24,25,27,28]. Indeed, all these studies were designed to assess O3FA as a therapeutic tool against COVID-19, whereas the present study was designed as a pragmatic clinical trial involving critically ill SARS-CoV-2 patients receiving PN. It is noteworthy that patients in the control group (Group I) received an average of 10% of their lipid intake as O3FA, whereas those in Groups II and III received an average of 28% and 43%, respectively. Regarding the prior studies assessing O3FA in COVID-19, only two used parenteral O3FA (Smoflipid^®^ or Omegaven^®^) for 5 days, with lower doses (3 g/day or 10 g/day, respectively) than those used in our intervention groups [23,27]. However, these studies showed controversial results, showing significant effects on CRP and IL-1 with lower O3FA (Smoflipid^®^), whereas the results in the study with the higher dose of O3FA, using Omegaven^®^, did not show the same effect.

The severity and duration of SARS-CoV-2 are associated with the hyperinflammatory response through the cytokine storm [5]. The inflammatory parameters assessed in this study, including CRP, IL-6, TNF-α, IFN-γ, IL-1Ra, and the chemokine CXCL10, are key markers of this inflammatory dissemination. These markers are known to be elevated in COVID-19 patients, with levels typically rising as the disease progresses [29]. The effects on secondary inflammatory markers were consistent with those observed for CRP and IL-6. However, significant effects of O3FA were only seen in IL-1Ra and CXCL10, while TNF-α levels showed a slight decrease and INF-γ a slight increase. Similarly, in a prior study assessing parenteral O3FA among COVID-19 patients, no significant variation in TNF-α levels was observed [27]. However, the dose used in this study was lower than in Groups II and III in our study (10 g/day for 5 days). On the contrary, a recent meta-analysis assessing O3FA in critically ill patients found positive results regarding TNF-α levels with the administration of O3FA-containing PN [16]. Our results showed a significant decrease in cytokine CXCL10 in SARS-CoV-2 patients receiving high doses of O3FA. However, similar to CRP and IL-6 results, patients in Group III (with the highest O3FA dose) did not achieve a significant reduction. Nevertheless, these patients achieved a notable decrease in CXCL10 levels (from 1342.30 [735.94] to 663.15 [421.25] on day 10), whereas patients allocated in the control group showed a slight increase. The lack of statistical significance might be due to the small sample size, with nine samples analyzed on day 5, and five on day 10. Similarly, O3FA administered in patients allocated in Groups II and III appeared to stabilize IL-1Ra levels, as there were no significant changes observed on days 5 and 10, unlike in Group I, where IL-1Ra levels increased significantly. The potential mechanisms underlying the reduction in inflammatory markers observed in our study have been previously discussed. O3FA may regulate the immune response by inhibiting various aspects of inflammation, such as leukocyte chemotaxis, and the production of proinflammatory cytokines and eicosanoids, including prostaglandins and leukotrienes derived from the omega-6 fatty acid ARA [10,18]. Additionally, O3FA promotes the formation of specialized pro-resolving mediators (SPMs), which lead to the resolution of inflammation [7,11].

We examined the differences in liver parameters and TG levels among the three groups. There were no differences among the liver parameters assessed except for the evolution of GGT in Group III between days 1 and 5. Two previous studies examining the effect of O3FA on COVID-19 patients assessed liver parameters, but in AST and ALT levels [21,22]. However, consistent with our findings, these studies did not observe significant changes in liver parameters. According to our results and previous results, O3FA supplementation in COVID-19 patients indicated appears to be safe regarding potential hepatotoxicity. Indeed, the addition of O3FA to PN is currently a strategy to prevent liver alterations in liver parameters and cholestasis in patients receiving PN [30]. Regarding the evolution of TG levels, only patients in Group II showed a significant decline on day 10. However, it is noteworthy that a lower proportion of patients in Group III develop hypertriglyceridemia. Similarly, the use of O3FA-containing PN is also suggested to control TG levels in patients receiving PN [30,31]. In line with this point, patients in Group II experienced a significant decline in TG levels, and Group III showed a lower proportion of hypertriglyceridemia episodes.

Our findings showed no effect on mortality in patients receiving O3FA supplementation. However, recent meta-analysis found a significant decline in mortality among patients receiving O3FA (RR = 0.76; 95% CI, [0.61, 0.93]; *p* = 0.010) [25]. It is noteworthy that our patients were critically ill, with high APACHE scores and a high average mortality rate, representing a population different from those in previous studies [24,25]. Findings regarding ICU and hospital stay showed a significant decline in both for the groups receiving O3FA supplementation. Indeed, results from multivariate analysis indicated a dose-dependent effect. As reported for acute respiratory distress syndrome (ARDS), the effect on ICU and hospital LOS could be explained by the impact of O3FA on inflammatory markers in critically ill patients with ARDS [18]. Given our results about shortening ICU and hospital LOS, it might be reasonable that unfavorable results for Group III regarding CRP and IL-6 would be because of the different timing in this group of patients [26].

This study is a pragmatic clinical trial assessing the supplementation of O3FA in COVID-19 critically ill patients with PN. Therefore, it was designed to understand the efficacy of changing PN formulation in real clinical practice. However, our study had some limitations. Although we achieved the target sample size, follow-up losses and the short duration of monitoring for inflammatory markers indicate a need for confirmation in larger multicenter studies. The loss of frozen samples might limit the reliability of results for secondary inflammatory markers. However, this loss was randomized and should have equally affected all allocation groups. The short follow-up period for inflammatory markers may have influenced the ability to detect changes, especially for longer-term effects. The fact that higher supplementation did not show as much effect on inflammatory markers as Group II, despite patients in Group III showing a dramatic decline in ICU and hospital stay, also deserves to be assessed in future studies. Future studies should extend the follow-up period to better assess the long-term effects of O3FA supplementation on inflammatory markers and clinical outcomes. Additionally, larger, multicenter studies are necessary to validate our findings and enhance their generalizability.

## 5. Conclusions

In conclusion, O3FA supplementation in critically ill SARS-CoV-2 patients receiving PN improved inflammatory markers such as IL-6 and CRP. However, the anticipated benefits in these markers were not observed with the highest dose of O3FA. This suggests a likely complex dose–response relationship, considering other factors such as the timing of O3FA administration. Notably, O3FA supplementation led to significant reductions in CXCL10 levels, supporting the anti-inflammatory role of O3FA. Furthermore, higher O3FA supplementation was associated with improved clinical outcomes, including reduced ICU and hospital LOS. Therefore, in the future, larger, multicenter studies are needed to investigate associations between inflammatory marker control and clinical outcomes with different O3FA doses. 

## Figures and Tables

**Table 1:** Baseline data of all patients (intention-to-treat analysis).

Parameter	Group I—No O3FA added (N = 24)	Group II—0.1 g/Kg/d O3FA (N = 24)	Group II—0.2 G/kg/d O3FA (N = 21)	Group II+III (N = 45)
Age, years	63.2 (5.6)	59.1 (10.5)	57.6 (11.9)	58.3 (11.0)
BMI	30.2 (4.2)	31.0 (7.2)	30.2 (4.9)	30.6 (6.2)
Weight, Kg	88.7 (12.6)	89.9 (26.9)	83.8 (13.6)	87.1 (21.8)
Male, n (%)	22 (91.6)	19 (79.2)	17 (81.0)	36 (80.0)
Hospital stay before PN, days	11.9 (6.0)	11.1 (8.3)	10.3 (7.6)	10.7 (7.9)
ICU stay before PN, days	10.0 (6.6)	7.9 (6.4)	8.2 (7.1)	8.1 (6.7)
APACHE II at ICU admission	17.4 (5.2)	19.0 (5.8)	17.2 (4.9)	18.2 (5.4)
APACHE II at PN start	17.8 (5.5)	19.3 (6.1)	18.9 (3.3)	19.1 (4.9)
CRP, mg/L	12.36 (9.04)	13.28 (9.28) ^2^	8.34 (8.59) ^1^	10.97 (9.28)
IL-6, pg/mL	619.95 (1473.11)	929.41 (1841.55) ^2^	404.86 (1037.62) ^1^	684.62 (1527.14)
Total bilirubin, mg/dL	0.82 (0.38)	1.03 (1.13)	0.89 (1.12)	0.96 (1.11)
AP, U/dL	124.91 (92.23)	140.42 (116.97)	100.90 (60.53)	121.98 (96.0)
GGT, U/dL	211.09 (188.06)	273.42 (262.07)	176.05 (123.75)	227.98 (212.78)
Triglycerides, mg/dL	255.04 (109.33)	289.21 (152.30)	216.43 (79.61)	255.24 (127.89)

^1^ Significant difference compared to Group II. ^2^ Significant difference compared to Group III. BMI: Body Mass Index. Data expressed as mean (SD).

**Table 2:** Within-subject analysis of inflammatory markers.

Variable	Allocation Group	Day 1 to Day 5	Day 1 to Day 10
CRP, mg/L	Group I	10.95 (7.78)–9.54 (8.51) [n = 17]	10.15 (8.08)–13.97 (8.08) [n = 14]
	Group II	13.65 (9.46)–8.67 (8.03) [n = 23] *	13.23 (9.04)–10.28 (8.78) [n = 19] *
	Group III	9.04 (9.02)–8.91 (7.46) [n = 17]	9.40 (10.61)–14.80 (13.27) [n = 12]
	Group II+III	11.50 (9.53)–8.77 (7.78) [n = 40] *	11.65 (9.55)–11.98 (10.66) [n = 31]
IL-6, pg/mL	Group I	358.54 (707.48)–165.55 (233.32) [n = 17]	304.78 (673.72)–95.25 (103.72) [n = 14]
	Group II	894.60 (1874.85)–506.31 (1127.1) [n = 23] *	1095.61 (2085.00)–294.51 (645.20) [n = 19] *
	Group III	492.18 (1141.47)–376.18 (995.12) [n = 17]	325.80 (725.26)–426.32 (835.14) [n = 12]
	Group II+III	723.57 (1599.37)–451.00 (1061.66) [n = 40] *	768.22 (1676.38)–336.78 (706.69) [n = 31] *
IFN-γ, pg/mL	Group I	0.97 (1.11)–1.56 (1.91) [n = 9]	1.02 (1.11)–5.52 (7.65) [n = 8]
	Group II	1.37 (1.16)–2.10 (3.04) [n = 13]	2.19 (2.75)–1.63 (1.43) [n = 11]
	Group III	4.01 (4.00)–19.03 (41.48) [n = 9]	3.89 (3.92)–6.40 (9.14) [n = 5]
	Group II+III	2.45 (2.94)–9.02 (27.08) [n = 22]	2.72 (3.13)–3.12 (5.37) [n = 16]
TNF-α, pg/mL	Group I	21.37 (10.77)–18.33 (7.98) [n = 9]	18.82 (7.41)–23.03 (9.37) [n = 8]
	Group II	23.38 (19.57)–20.49 (14.75) [n = 13]	32.55 (33.42)–23.22 (16.92) [n = 11]
	Group III	22.97 (8.34)–22.88 (12.92) [n = 9]	26.73 (5.35)–26.72 (22.70) [n = 5]
	Group II+III	23.21 (15.67)–21.47 (13.76) [n = 22]	30.73 (27.57)–24.31 (18.19) [n = 16]
IL-1Ra, pg/mL	Group I	1745.44 (816.75)–3398.63 (2242.79) [n = 9] *	1706.25 (727.72)–4599.13 (2984.13) [n = 8] *
	Group II	4849.01 (5990.47)–4040.12 (3411.48) [n = 13]	5688.03 (6511.04)–5302.14 (5644.86) [n = 11]
	Group III	3024.61 (2031.39)–4343.72 (4416.36) [n = 9]	3906.40 (2095.45)–4304.20 (5615.06) [n = 5]
	Group II+III	4102.66 (4787.59)–4164.32 (3755.52) [n = 22]	5131.27 (5491.88)–4990.28 (5466.31) [n = 16]
CXCL10, pg/mL	Group I	448.33 (272.87)–522.26 (410.64) [n = 9]	418.50 (224.66)–515.68 (322.15) [n = 8]
	Group II	757.05 (682.09)–380.50 (161.87) [n = 13] *	963.27 (786.52)–435.05 (231.70) [n = 11] *
	Group III	1155.71 (826.90)–689.61 (563.26) [n = 9]	1342.30 (735.94)–663.15 (421.25) [n = 5]
	Group II+III	920.14 (752.72)–506.95 (400.04) [n = 22] *	1081.71 (767.96)–506.33 (308.28) [n = 16] *

* *p* < 0.05. Data expressed as mean (SD).

**Table 3:** Caloric and lipid intake, days of PN, and secondary clinical outcomes.

Parameter	Group I No O3FA added (N = 17)	Group II 0.1 g/Kg/d O3FA (N = 23)	Group II 0.2 G/kg/d O3FA (N = 17)	Group II+III (N = 40)
PN days	17.76 (7.51)	16.74 (11.97)	13.29 (6.44)	16.48 (10.94)
APACHE II score ICU admission	17.41 (5.17)	19.30 (5.68)	16.82 (4.65)	18.25 (5.35)
APACHE II score PN start	17.11 (5.59)	19.52 (6.13)	19.00 (3.55)	19.30 (5.14)
kcal/day	1553.85 (111.12)	1673.98 (928.70)	1476.85 (145.88)	1590.20 (710.63)
Lipid g/day	47.59 (10.91)	50.94 (30.76)	48.82 (9.53)	50.04 (23.92)
O3FA g/day	4.81 (1.75)	14.33 (3.28)	21.30 (4.09)	17.29 (5.01)
O3FA g/kg/day	0.05 (0.02) ^2,3^	0.16 (0.03) ^1,3^	0.26 (0.06) ^1,3^	0.20 (0.06)^1^
Kcal/kg/day (day 5)	15.62 (2.43)	15.25 (3.93)	16.75 (2.45)	15.89 (3.43)
Lipid g/kg/day (day 5)	0.49 (0.16)	0.46 (0.17)	0.54 (0.13)	0.50 (0.16)
O3FA g/day (day 5)	3.36 (1.70)	13.65 (2.83)	21.83 (5.50)	17.13 (5.81)
Kcal/kg/day (day 10)	16.42 (9.92)	15.28 (203.98)	17.67 (2.75)	16.24 (3.40)
Lipid g/kg/day (day 10)	0.50 (0.17)	0.46 (0.16)	0.57 (0.14)	0.50 (0.16)
O3FA g/day (day 10)	4.70 (1.57) ^2,3^	15.25 (3.54) ^1,3^	21.95 (4.74) ^1,3^	17.93 (5.20) ^1^
Secondary Outcomes (Per-Protocol)	Group I (N = 24)	Group II (N = 24)	Group III (N = 19)	Group II+III (N = 43)
Hospital LOS, days	74.83 (55.46) ^3,2+3^	56.60 (34.18) *	31.42 (11.86) ^1^	45.41 (29.18) ^1^
ICU LOS, days	38.89 (24.99) ^3,2+3^	32.67 (20.90) *	15.92 (5.82) ^1^	25.22 (17.93) ^1^
Mortality, n (%)	3 (25.00)	9 (37.50)	7 (36.84)	16 (37.21)
Secondary Outcomes (Intention-to-Treat)	Group I (N = 24)	Group II (N = 24)	Group III (N = 21)	Group II+III (N = 45)
Hospital LOS, days	74.83 (55.46) ^3,2+3^	56.60 (34.18)	38.38 (27.57) ^1^	48.14 (32.01) ^1^
ICU LOS, days	38.89 (24.99) ^3,2+3^	32.67 (20.90)	19.85 (15.23) ^1^	25.58 (17.43) ^1^
Mortality, n (%)	6 (25.00)	9 (39.13)	8 (38.10)	17 (37.78)

^1^ Significant difference compared to Group I. ^2^ Significant difference compared to Group II. ^3^ Significant difference compared to Group III. ^2+3^ Significant difference compared to Group II+III. * *p* = 0.07 (difference compared to Group I). Data expressed as mean (SD).

**Table 4:** Within-subject analysis of liver parameters and triglycerides levels.

Variable	Allocation Group	Day 1 to Day 5	Day 1 to Day 10
TB, mg/dL	Group I	0.84 (0.35)–1.08 (0.98) [n = 17]	0.84 (0.35)–1.33 (1.31) [n = 14]
	Group II	1.06 (1.14)–1.03 (1.21) [n = 23]	1.04 (1.15)–0.84 (0.48) [n = 18]
	Group III	0.67 (0.32)–0.82 (0.69) [n = 17]	0.72 (0.35)–0.93 (0.79) [n = 12]
AP, U/dL	Group I	140.5 (105.81)–166.19 (141.47) [n = 17]	114.31 (75.41)–140.92 (112.98) [n = 14]
	Group II	141.30 (119.52)–152.04 (150.85) [n = 23]	136.94 (130.39)–149.17 (168.34) [n = 18]
	Group III	108.94 (64.16)–135.65 (93.38) [n = 17]	107.83 (62.14)–127.42 (50.11) [n = 12]
GGT, U/dL	Group I	235.31 (217.66)–317.38 (246.88) [n = 17]	217.85 (224.41)–281.15 (189.83) [n = 14]
	Group II	275.30 (267.79)–275.30 (207.45) [n = 23]	271.67 (289.13)–241.94 (161.18) [n = 18]
	Group III	180.18 (131.96)–317.59 (348.30) [n = 17] *	190.67 (122.08)–304.67 (219.33) [n = 12]
TG, mg/dL	Group I	282.88 (112.79)–292.65 (114.05) [n = 17]	291.43 (118.65)–286.07 (96.38) [n = 14]
	Group II	277.13 (143.50)–264.52 (111.47) [n = 23]	297.28 (145.95)–244.22 (95.46) [n = 18] *
	Group III	222.41 (81.08)–225.76 (101.35) [n = 17]	219.42 (91.53)–238.58 (139.39) [n = 12]

* *p* < 0.05. Data expressed as mean (SD).

**Table 5:** Multivariant regression models for mortality and ICU and hospital LOS.

Outcome	Variable	Odds Ratio (CI 95%)
Mortality (ITT)	APACHE II	1.28 (1.10–1.48) *
	IL-6 (day 1)	1.00 (1.00–1.00)
Mortality (PP)	APACHE II	1.27 (1.10–1.47) *
	IL-6 (day 1)	1.00 (1.00–1.00)
Outcome	Variable	Coefficient (CI 95%)
ICU LOS (ITT)	APACHE II	1.36 (0.05–2.67) *
	Age (years)	0.58 (0.03–1.14) *
	PN days	0.97 (0.36–1.58) *
ICU LOS (ITT)	APACHE II	1.58 (0.24–2.93) *
	PN days	0.71 (0.13–1.29) *
	O3FA g/kg/day	−81.25 (−146.28 to −16.23) *
Hospital LOS (ITT)	PN days	1.43 (0.14–2.72) *
	Group II	−22.59 (−51.56–6.37)
	Group III	−29.70 (−60.19 to −0.78) *
Hospital LOS (ITT)	PN days	1.38 (0.15–2.60) *
	O3FA g/kg/day	−147.26 (−284.57 to −9.94) *
ICU LOS (PP)	APACHE II	1.17 (−0.05 to 2.38)
	PN days	0.80 (0.22–1.38) *
	Group II	−9.22 (−21.99–3.54)
	Group III	−19.97 (−33.67 to −6.28) *
ICU LOS (PP)	APACHE II	1.19 (−0.01–2.40)
	PN days	0.83 (0.28 –1.38) *
	O3FA g/kg/day	−91.09 (−152.08 to −30.09)*
Hospital LOS (PP)	PN days	1.61 (0.38–2.83) *
	Group II	−23.12 (−50.36–4.12)
	Group III	−36.77 (−65.98 to −7.56) *
Hospital LOS (PP)	PN days	1.60 (0.43–2.77) *
	Total O3FA g/kg/day	−167.6 (−298.67 to −37.05) *

* *p* < 0.05. CI: Confidence Interval.

## Data Availability

The data presented in this study are available on request from the corresponding author. The data are not publicly available due to privacy reasons.

## Supplementary Materials

The following supporting information can be downloaded at: https://www.mdpi.com/article/10.3390/nu16183046/s1, Table S1: Differences between groups on day 5 and day 10.
