# Supplementary material for: Impact of Omega-3 Fatty Acid Supplementation in Parenteral Nutrition on Inflammatory Markers and Clinical Outcomes in Critically Ill COVID-19 Patients: A Randomized Controlled Trial
Source: Nutrients. 2024 Sep 10;16(18):3046. doi: 10.3390/nu16183046 (PMC11434828; doi:10.3390/nu16183046)
Supplement: Supplementary file 1 [file nutrients-16-03046-s001.zip › nutrients-3186091-supplementary.pdf]

Supplementary Table

Supplementary Table S1. Differences between groups on day 5 and day 10.

Parameter	Group I - No O3FA added	Group II - 0.1 g/Kg/d O3FA	Group II - 0.2 G/kg/d O3FA	Group II+III
**Day 5** CRP, mg/L	9.54 (8.51)	8.67 (8.03)	8.70 (7.31)	10.97 (9.28)
IL-6, pg/mL	184.34 (245.09)	506.31 (1127.10)	353.15 (941.62)	684.62 (1527.14)
IFN-γ, pg/mL	1.43 (1.85)*	2.15 (2.93)	19.04 (41.48)*	8.76 (26.49)
TNF-α, pg/mL	17.16 (8.38)	20.25 (14.20)	22.88 (12.92)	21.28 (13.47)
IL-1Ra, pg/mL	3154.97 (2250.54)	3940.82 (3298.63)	4343.72 (4416.36)	4108.42 (4714.24)
CXCL10, pg/mL	487.83 (402.17)	367.46 (162.99)	689.61 (563.26)	493.52 (396.12)
Total bilirubin, mg/dL	1.08 (0.98)	1.03 (1.21)	1.05 (1.27)	1.03 (1.22)
AP, U/dL	166.19 (141.47)	152.04 (150.85)	128.16 (91.22)	152.81 (114.07)
GGT, U/dL	317.38 (246.88)	275.30 (207.45)	308.58 (330.62)	265.55 (182.68)
Triglycerides, mg/dL	292.65 (114.05)	264.52 (111.47)	225.00 (96.09)	236.97 (114.32)
**Day 10** CRP, mg/L	13.97 (13.52)	10.19 (8.54)	14.80 (13.27)	11.98 (10.66)
IL-6, pg/mL	95.25 (103.72)	279.67 (630.35)	426.32 (835.14)	336.44 (706.69)
IFN-γ, pg/mL	4.93 (7.37)	1.56 (1.38)	4.67 (8.03)	2.70 (5.00)
TNF-α, pg/mL	21.48 (9.91)	23.81 (16.26)	21.53 (20.54)	22.97 (17.42)
IL-1Ra, pg/mL	4158.67 (3088.35)	5041.38 (5457.44)	3430.14 (4825.29)	4447.76 (5157.51)
CXCL10, pg/mL	472.49 (328.02)	406.79 (241.63)	531.11 (11)	452.59 (310.26)
Total bilirubin, mg/dL	1.33 (1.31)	0.97 (0.72)	1.05 (1.27)	1.00 (1.22)
AP, U/dL	137.93 (90.39)	145.79 (113.55)	163.92 (119.04)	152.81 (114.07)
GGT, U/dL	297.50 (192.36)	240.84 (156.71)	304.67 (219.33)	265.55 (182.68)
Triglycerides, mg/dL	286.07 (96.38)	235.95 (99.54)	238.58 (139.39)	236.97 (114.32)

* Differences close to significant (p= 0.06). Data expressed as mean (SD)
